# Clinical significance of early recurrence of atrial fibrillation after cryoballoon vs. radiofrequency ablation—A propensity score matched analysis

**DOI:** 10.1371/journal.pone.0219269

**Published:** 2019-07-02

**Authors:** Michifumi Tokuda, Seigo Yamashita, Seiichiro Matsuo, Mika Kato, Hidenori Sato, Hirotsuna Oseto, Eri Okajima, Hidetsugu Ikewaki, Masaaki Yokoyama, Ryota Isogai, Kenichi Tokutake, Kenichi Yokoyama, Ryohsuke Narui, Shin-ichi Tanigawa, Michihiro Yoshimura, Teiichi Yamane

**Affiliations:** Department of Cardiology, The Jikei University School of Medicine, Tokyo, Japan; Karolinska Institutet, SWEDEN

## Abstract

**Objectives:**

One of the mechanisms of early recurrence of atrial fibrillation (ERAF) after AF ablation is considered to be the inflammatory reaction of the atrial tissue. The aim of this study is to compare the clinical significance of ERAF at each stage for true AF recurrence between cryoballoon (CB) and radiofrequency (RF) ablation.

**Methods:**

Among 798 paroxysmal AF patients who underwent an initial ablation, 460 patients (CB, n = 230; RF, n = 230) were selected by propensity score matching. Very ERAF (VERAF), ERAF-1M, ERAF-3M and true AF recurrence were defined as AF recurrence at 0–2, 3–30, 31–90 days and more than 90 days after the procedure, respectively.

**Results:**

The patient characteristics of the two groups were similar. ERAF was observed 21% and 27% in the CB and RF groups, respectively. In both the CB and RF group, VERAF, ERAF-1M and ERAF-3M were more frequently observed in patients with true AF recurrence than in those without. In a multivariable analysis, ERAF-1M and ERAF-3M were found to be independent predictors of true AF recurrence in both the CB (P = 0.04 and P<0.001, respectively) and RF groups (P = 0.02 and P = 0.001, respectively). However, while VERAF was associated with true AF recurrence after RF ablation (P = 0.03), it was not associated with true AF recurrence after CB ablation (P = 0.19).

**Conclusion:**

The relationship between ERAF and true AF recurrence differed between the RF and CB ablation groups. While VERAF was associated with true AF recurrence after RF ablation, it was not a predictor of true AF recurrence after CB ablation.

## Introduction

Pulmonary vein isolation (PVI) is the cornerstone of catheter ablation for atrial fibrillation (AF), especially in paroxysmal AF patients [1]. Cryoballoons (CBs) have proven to be an effective tool for PVI in patients with AF. Recent randomized trials have shown the noninferiority of CB ablation to radiofrequency (RF) ablation with respect to the treatment efficacy in patients with drug-refractory paroxysmal AF [2, 3].

The recurrence AF is observed at each stage after catheter ablation of AF. One of mechanisms of early recurrence of AF (ERAF) is considered to be a transient phenomenon due to the inflammatory reaction of atrial tissue. Thus, atrial tachyarrhythmias occurring during the first 3 months after ablation are not counted as a “true” recurrence [4, 5]. Because of the significant associations between ERAF and late AF recurrence after RF [4] or CB [6–8] ablation, and the difference in the inflammatory reactions observed immediately after and months after ablation, the definition of the blanking period should be reconsidered. The increase in the levels of Troponin I and Creatine Kinase after CB ablation was significantly higher in comparison to after RF ablation [9], suggesting that the processes and grades of inflammatory reaction differ between CB and RF ablation. The clinical significance of ERAF in each stage during the blanking period may differ between CB and RF ablation. The aim of this study is to compare the clinical significance of ERAF at each stage for true AF recurrence between CB and RF ablation.

## Methods

### Study subjects

The study population included 798 consecutive patients who had undergone an initial catheter ablation procedure for paroxysmal AF in our institute. The decision to perform RF ablation or CB ablation was based on the preference of each individual operator. All of the patients underwent enhanced computed tomography (CT) prior to ablation to establish the anatomy of the LA and PV. Antiarrhythmic drugs were discontinued for at least five half-lives prior to ablation. Transesophageal echocardiography was performed prior to the procedure to rule out the presence of LA thrombus. Paroxysmal AF was defined as AF that spontaneously terminated within seven days. Each patient provided their written informed consent. The study and data collection were performed in accordance with protocols that had been approved by the Human Research Committee of the Jikei University School of Medicine.

### Radiofrequency ablation

RF-PVI was performed as previously described under institutional approval [10, 11]. PV mapping was performed with a 20-polar circular catheter (Lasso; Biosense Webster, Diamond Bar, CA, USA). All four PVs were individually targeted for disconnection from the LA. RF energy was delivered at the PV antrum using an open irrigated-tip ablation catheter with a power limit of 25–35 W. The endpoint of the PVI was the establishment of a bidirectional conduction block between the LA and the PV. The exit block was confirmed by pacing inside the PV with a Lasso catheter. The absence of acute conduction recovery between the PVs and the LA was confirmed in each PV after waiting at least 30 minutes after the final RF application.

### Cryoballoon ablation

CB ablation was performed as previously reported. [12] A single transseptal puncture was performed using an RF needle (Baylis Medical, Montreal, QC, Canada) and an 8-Fr long sheath (SL0, AF Division, St. Jude Medical). The transseptal sheath was exchanged over a guide wire for a 15-Fr steerable sheath (Flexcath Advance; Medtronic, Minneapolis, MN, USA). A 20–30 mm circular mapping catheter (Lasso; Biosense Webster) was used to map all of the PVs before and after the CB to confirm electrical isolation. PVI was performed with a single balloon technique using a second-generation CB (Arctic Front Advance; Medtronic). A 28-mm CB catheter was used in all of the patients. A spiral mapping catheter (Achieve; Medtronic) was used to advance the CB and map the PV potentials. Complete sealing at the antral aspect of the PV was confirmed by the injection of contrast medium. To avoid phrenic nerve injury, the diaphragmatic compound motor action potentials (CMAPs) were monitored during phrenic nerve pacing during each CB application. The procedural endpoint was defined as the establishment of a bidirectional PV-LA block, which was verified by a 20–30 mm circular mapping catheter. If electrical isolation was not achieved after a total of 3 CB applications (180 seconds for each application) per vein, additional touch-up ablation was performed with a conventional RF or cryothermal (Freezer Max; Medtronic) catheter.

### Induction and elimination of dormant PV conduction

After waiting for at least 30 minutes after the final RF or CB application for PVI, ATP (20 mg) was rapidly intravenously administered to induce dormant PV conduction under the continuous infusion of isoproterenol (20 μg/min) in all patients. If dormant PV conduction was provoked, additional RF or cryothermal energy was applied at the earliest transient PV activation site identified by the Lasso catheter in order to eliminate the dormant PV conduction.

### Patient follow-up

No antiarrhythmic drugs were prescribed after the procedure. The patients underwent continuous, in-hospital ECG monitoring for two days after the procedure. The patients were then carefully observed (two weeks after discharge, then every month thereafter) at the cardiology clinic. The outcome of AF ablation was evaluated based on the patient’s symptoms, the ECG findings at periodic follow-up examinations and periodic 24-hour ambulatory monitoring (at 1, 3, 6, 9, and 12 months after performing the procedure). In patients who had palpitations, but could not be detected by routine examinations, a 5-day event recorder or portable ECG recorder (HeartScan, Omron Healthcare, Kyoto, Japan) was used. The recurrence of AF was defined as AF lasting for more than 30 seconds. Very ERAF (VERAF), ERAF-1M, ERAF-3M and true AF recurrence were defined as AF recurrence at 0–2, 3–30, 31–90 days and more than 90 days after the procedure, respectively.

### Statistical analysis

Propensity scores were calculated for each of the 798 patients based on a multivariable logistic regression model. A total of 13 characteristics (all variables in Table 1) hypothesized to be associated with the outcomes of catheter ablation were assessed for inclusion in the model as independent variables. All 13 characteristics were retained in the model with stepwise selection and were subsequently used to generate propensity scores. In the selection process, a P value of 0.05 was used as a cutoff for a characteristic to be entered and remain in the model. In order of stepwise selection, the matching variables were as follows: sex, age, body mass index, LA diameter, left ventricular ejection fraction, estimated glomerular filtration rate, B-type natriuretic peptide, history of AF, hypertension, diabetes mellitus, congestive heart failure, number of anti-arrhythmic drugs and CHADS_2_ score. The baseline characteristics after matching were assessed by evaluating the standardized mean differences; a standardized mean difference numerically <0.1 was considered to be acceptable. Based on the propensity score, the patients who underwent CB-PVI and RF-PVI patients were matched on a 1:1 basis with a nearest neighbor algorithm without replacement (caliper width: 1/5 logit of the standard deviation).

**Table 1:** Patient characteristics.

	Cryoballoon ablation (n = 230)	Radiofrequency ablation (n = 230)	*P* value
Sex (male)	190 (83%)	197 (86%)	0.33
Age (years)	58.9 ± 9.6	58.7 ± 10.4	0.85
Body mass index (kg/m^2^)	24.0 ± 3.2	23.8 ± 3.2	0.44
LA diameter (mm)	37.6 ± 5.6	37.0 ± 5.0	0.25
LV ejection fraction (%)	63.6 ± 5.0	64.5 ± 5.0	0.13
eGFR (ml/min/1.73m^2^)	74.5 ± 14.6	76.7 ± 16.4	0.19
BNP (pg/ml)	50.3 ± 69.0	48.9 ± 68.0	0.83
History of AF	3.5 ± 4.8	3.1 ± 3.7	0.33
Hypertension	103 (45%)	84 (37%)	0.07
Diabetes mellitus	25 (11%)	21 (9%)	0.33
CHADS_2_ Score	0.7 ± 0.8	0.6 ± 0.8	0.20
No. of AADs	1.0 ± 0.7	1.0 ± 0.8	0.92

The data are presented as the mean ± SD or n (%). LA, left atrium; LV, left ventricle; eGFR, estimated glomerular filtration rate; BNP, B-type natriuretic peptide; AADs, anti-arrhythmic drugs.

Continuous variables were expressed as the mean ± standard deviation. An unpaired Student’s *t*-test or the Mann-Whitney U test was used for the comparison of continuous variables. Categorical variables, expressed as numbers or percentages, were analyzed using the chi-squared test unless the expected value in any cell was less than 5, in which case Fisher’s exact test was used. P values of <0.05 were considered to indicate statistical significance. Survival curves were created using the Kaplan-Meier method and comparisons between groups were performed using a log-rank test. To identify factors independently associated with true AF recurrence, univariate factors with P values of <0.10 were analyzed using a Cox proportional hazard regression model (multivariate analysis). All statistical analyses were performed using the SPSS software program (version 21.0.0; SPSS, Chicago, IL, USA).

## Results

### Study population

CB and RF ablation were performed in 244 and 544 patients, respectively. The resulting propensity score-matched data set included 460 patients (CB-PVI, n = 230; RF-PVI, n = 230). The receiver operating characteristic curve area (c-statistic) of the propensity-score model was 0.75. After propensity score matching, there were no significant differences between the two groups with regard to the baseline patient characteristics. (Table 1) The total procedure time in the patients who underwent CB ablation was significantly shorter in comparison to those who underwent RF ablation (140±32 vs. 173±49 min, P = 0.001). In the CB group, CB-PVI failed in 9% of the PVs, which necessitated touch-up ablation. The incidence of major complications in the CB and RF groups did not differ to a statistically significant extent (4.3% vs. 2.6%; P = 0.31).

### AF recurrence after the ablation procedure

**Fig 1:**
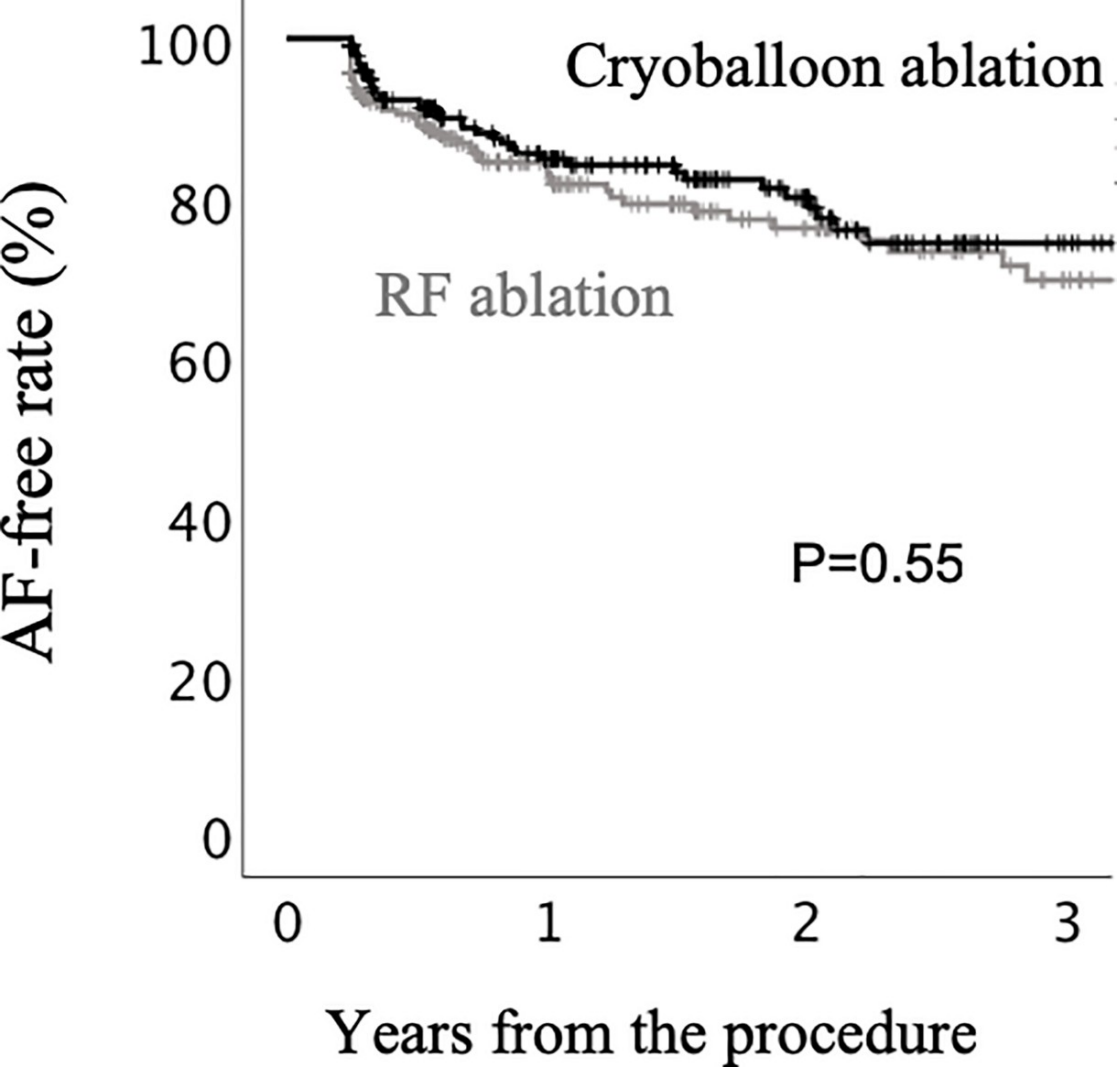
The AF-free rate after initial cryoballoon (CB) or radiofrequency (RF) ablation. Kaplan-Meier curves showing the AF-free rate after the initial CB and RF catheter ablation procedure.

After the initial ablation procedure, the incidence of ERAF (0–90 days) was similar between the CB and RF groups (21% vs. 27%, P = 0.15, Table 2). VERAF (0–2 days) and ERAF-3M (31–90 days) were similarly observed between the CB and RF groups (14% vs. 11%, P = 0.33 and 8.3% vs.8.7%, P = 0.87, respectively). On the other hand, ERAF-1M (3–30 days) was less frequently observed in the CB group than in the RF group (5.7% vs. 11%, P = 0.04). In the CB group, the incidence of VERAF was higher than that of ERAF-1M (14% vs. 5.7%, P = 0.002). At a mean of 20.1 ± 12.7 months after the initial ablation procedure, there was no marked difference in the AF-free rates between the two groups (P = 0.55, Fig 1). No patients died during the follow-up period.

**Table 2:** Incidence of AF recurrence.

	Radiofrequency ablation (n = 230)	Cryoballoon ablation (n = 230)	*P* value
All ERAF (0–90 days)	61 (27%)	48 (21%)	0.15
Very ERAF (0–2 days)	26 (11%)	33 (14%)	0.33
ERAF-1M (3–30 days)	25 (11%)	13 (5.7%)	0.04
ERAF-3M (31–90 days)	20 (8.7%)	19 (8.3%)	0.87
True recurrence of AF (from 91 days)	49 (21%)	38 (17%)	0.19

ERAF indicates early recurrence of atrial fibrillation.

### Patients who underwent cryoballoon ablation

True AF recurrence occurred in 38 (17%) patients after CB ablation. Table 3 compares the characteristics of patients with and without true AF recurrence. In the patients with true AF recurrence after CB ablation, the BNP level (70.2 ± 97.2 vs. 44.4 ± 59.4 pg/ml, P = 0.04) and CHADS_2_ score (0.9 ± 1.0 vs. 0.5 ± 0.7, P = 0.04) were higher and VERAF (32% vs. 11%, P = 0.001), ERAF-1M (24% vs. 2.1%, P<0.001) and ERAF-3M (34% vs. 3.1%, P<0.001) were more frequently observed in comparison to patients without true AF recurrence. In the multivariate analysis, while ERAF-1M (HR = 2.651, 95% CI = 1.060–6.628, P = 0.04) and ERAF-3M (HR = 4.931, 95% CI = 2.177–11.169, P<0.001) were associated with true AF recurrence, VERAF was not (HR = 1.628, 95% CI = 0.791–3.352, P = 0.19) (Table 4).

**Table 3:** Comparison of the cases with and without true AF recurrence after cryoballoon ablation.

	AF recurrence (-) N = 192	AF recurrence (+) N = 38	*P*
Sex (male)	166 (87%)	31 (82%)	0.43
Age (years)	58.5 ± 10.5	60.0 ± 10.5	0.39
Body mass index (kg/m^2^)	23.8 ± 3.1	23.9 ± 3.4	0.91
LA diameter (mm)	37.8 ± 4.8	37.9 ± 5.9	0.34
LV ejection fraction (%)	64.6 ± 5.0	64.0 ± 5.0	0.53
eGFR (ml/min/1.73m^2^)	77.5 ± 17.4	73.6 ± 11.2	0.16
BNP (pg/ml)	44.4 ± 59.4	70.2 ± 97.2	0.04
History of AF	3.0 ± 3.6	4.0 ± 3.9	0.15
Hypertension	68 (35%)	16 (42%)	0.43
Diabetes mellitus	16 (8.3%)	5 (13%)	0.35
CHADS_2_ Score	0.5 ± 0.7	0.9 ± 1.0	0.04
No. of AADs	1.0 ± 0.7	1.0 ± 0.8	0.72
Total procedure time (min)	139 ± 33	146 ± 28	0.20
All ERAF (0–90 days)	25 (13%)	23 (61%)	<0.001
Very ERAF (0–2 days)	21 (11%)	12 (32%)	0.001
ERAF-1M (3–30 days)	4 (2.1%)	9 (24%)	<0.001
ERAF-3M (31–90 days)	6 (3.1%)	13 (34%)	<0.001

The data are presented as the mean ± SD or n (%). The abbreviations are the same as in the previous tables.

**Table 4:** Predictors of true AF recurrence after cryoballoon ablation.

	Multivariable analysis		
	Odds ratio	95%CI	P value
BNP	1.001	0.009–1.005	0.50
CHADS_2_ score	1.328	0.919–1.920	0.13
Very ERAF	1.628	0.791–3.352	0.19
ERAF-1M	2.651	1.060–6.628	0.04
ERAF-3M	4.931	2.177–11.169	<0.001

The abbreviations are the same as in the previous tables.

### Patients who underwent RF ablation

In the RF group, true AF recurrence was observed in 49 (21%) patients. The serum BNP levels (81.8 ± 120.0 vs. 42.2 ± 44.7 pg/ml, P = 0.001) were higher and VERAF (18% vs. 9.3%, P<0.001), ERAF-1M (27% vs. 7.1%, P = 0.001) and ERAF-3M (33% vs. 2.2%, P<0.001) were more frequently observed in the patients with true AF recurrence than those without (Table 5). In the multivariate analysis, the serum BNP level (HR = 1.003 95%CI = 1.000–1.005, P = 0.02), VERAF (HR = 2.483, 95% CI = 1.121–5.502, P = 0.03), ERAF-1M (HR = 2.486, 95% CI = 1.165–5.305, P = 0.02) and ERAF-3M (HR = 5.554, 95% CI = 2.652–11.628, P<0.001) were associated with true AF recurrence (Table 6). It is noteworthy that while VERAF (0–2 days) was associated with true AF recurrence after RF ablation, it was not a predictor of true AF recurrence after CB ablation.

**Table 5:** Comparison between the patients with and without true AF recurrence after RF ablation.

	AF recurrence (-) N = 181	AF recurrence (+) N = 49	*P*
Sex (male)	149 (82%)	41 (84%)	0.83
Age (years)	58.7 ± 9.9	59.6 ± 8.7	0.54
Body mass index (kg/m^2^)	24.1 ± 3.2	23.8 ± 2.9	0.44
LA diameter (mm)	37.6 ± 5.4	37.5 ± 6.6	0.90
LV ejection fraction (%)	63.8 ± 6.9	62.8 ± 7.1	0.42
eGFR (ml/min/1.73m^2^)	75.7 ± 14.0	69.9 ± 15.8	0.14
BNP (pg/ml)	42.2 ± 44.7	81.8 ± 120.0	0.001
History of AF	3.3 ± 4.4	4.4 ± 5.8	0.23
Hypertension	79 (44%)	24 (49%)	0.51
Diabetes mellitus	22 (12%)	3 (6.1%)	0.23
CHADS_2_ Score	0.7 ± 0.8	0.6 ± 0.7	0.69
No. of AADs	1.0 ± 0.7	0.9 ± 0.7	0.81
Total procedure time (min)	173 ± 48	176 ± 54	0.001
All ERAF (0–90 days)	32 (18%)	29 (59%)	<0.001
Very ERAF (0–2 days)	17 (9.3%)	9 (18%)	<0.001
ERAF-1M (3–30 days)	12 (7.1%)	13 (27%)	0.001
ERAF-3M (31–90 days)	4 (2.2%)	16 (33%)	<0.001

The data are presented as the mean ± SD or n (%). The abbreviations are the same as in the previous tables.

**Table 6:** Predictors of true AF recurrence after RF ablation.

	Odds ratio	95%CI	P value
BNP	1.003	1.000–1.005	0.02
Very ERAF	2.483	1.121–5.502	0.03
ERAF-1M	2.486	1.165–5.305	0.02
ERAF-3M	5.554	2.652–11.628	0.001

The abbreviations are the same as in the previous tables.

## Discussion

### Major findings

The major findings of this study are as follows: (1) while VERAF (0–2 days) and ERAF-3M (31–90 days) were similarly observed between the CB and RF group, ERAF-1M (3–30 days) was less frequently observed in the CB group than the RF group; (2) ERAF-1M and ERAF-3M were independent predictors of true AF recurrence in both the CB and RF groups; and (3) while VERAF was associated with true AF recurrence after RF ablation, VERAF was not associated with true AF recurrence after CB ablation. To the best of our knowledge, this is the first study to divide the three-month blanking period after AF ablation into 3 groups and compare the predictors of true AF recurrence between patients undergoing CB and RF ablation.

### Early recurrence of AF after RF ablation

The incidence of ERAF has been reported to range from 16% to 65% regardless of the ablation energy source. [13] Since 32–59% of patients with ERAF did not continue to experience true AF recurrence [4], expert consensus documents stated that ERAF (0–90 days) after an ablation procedure does not count as true AF recurrence and that a blanking period of three months should be employed after ablation [14]. However, in this study ERAF at every stage after RF ablation associated to the true AF recurrence. As in our study, many studies had reported a significant association between ERAF and true AF recurrence after RF ablation [4, 15–18]. Thus, the clinical significance of ERAF to true AF recurrence is still controversial. Pending clinical trial regarding to head-to-head comparisons of blanking periods, earlier repeat ablation cannot be routinely recommended. Treatment decisions should be made on case-by-case basis. In highly symptomatic patients with multiple atrial tachyarrhythmia recurrences, it may be reasonable to perform a repeat ablation procedure before the end of the 90-day blanking period.

The mechanism of true AF recurrence is considered to involve the reconnection of the isolated PV, untreated or newly developed non-PV foci, and incomplete ablation. On the other hand, ERAF was highly influenced by a post-procedural inflammatory reaction of the atrial tissue, maturation of ablation lesions, or imbalance of the autonomic system. Since the post-procedural inflammatory reaction gradually degrades over time after the procedure, there must be differences in the involvement of the inflammatory reaction in AF recurrence immediately after and months after the procedure. Previous studies have reported controversial results regarding the characteristics and significance of VERAF after RF ablation of AF. Chang et al. [19] reported that VERAF (0–2 days) in patients with paroxysmal AF is not associated with long-term recurrence. On the other hand, a previous study reported that VERAF (0–3 days) was associated with long-term recurrence [20]. In the present study, VERAF (0–2 days) was independently associated with true AF recurrence after RF ablation.

### Early recurrence of AF after CB ablation

In previous studies, ERAF after CB ablation was observed in 17–52% of patients [8, 21–23] and was strongly associated with true AF recurrence [8, 21]. The incidence of ERAF after CB ablation can vary according to the follow-up method. The most reliable study with subsequent implantation of an implantable loop recorder after CB ablation reported the incidence of ERAF was 28% [8]. In the present study, ERAF was observed in 21% of the patients after CB ablation; this rate was consistent with previous studies.

While the rates of VERAF and ERAF-1M were similar in the RF group, the incidence of VERAF was higher than that of ERAF-1M in the CB group (P = 0.002). CB ablation results in the creation of dense, well-demarcated homogeneous lesions through a directed freezing process, suggesting that the myocardial injury after CB ablation may be more extensive than that which occurs after RF ablation. The observation of higher levels of myocardial enzymes after CB ablation in comparison to RF ablation supports this hypothesis [9]. The extensive myocardial injury after CB ablation can explain the higher incidence of VERAF and why it was not associated with the recurrence of AF in the CB group. Budzianowski et al. reported that the elevation of CK-MB and Troponin-T after CB ablation were independent predictors of ERAF following CB ablation.[23] As previous studies have shown, ERAF was still an important predictive factor of true AF recurrence. However, VERAF after CB ablation had no impact on the incidence of true AF recurrence and can be considered a transient phenomenon. The definition of blanking period after the ablation procedure should therefore be reconsidered according to the type of ablation device that is used.

## Study limitations

The present study was associated with several limitations. First, the study employed a non-randomized, observational design and was performed in a single center. Even with the propensity score-matched analysis, a selection bias cannot be excluded. In this study some cases of recurrent AF might have been missed, as several tools were used to detect recurrence, including ECG at periodic follow-ups, a 5-day event recorder, a portable ECG recorder and periodic ambulatory monitoring. Further studies using an implantable loop recorder are recommended to more precisely evaluate the incidence of recurrent AF after RF and CB ablation.

## Conclusions

The relationship between ERAF and true AF recurrence differed between RF and CB ablation. ERAF-1M and ERAF-3M were the independent predictors of true AF recurrence in both the CB and RF groups. Although VERAF (0–2 days) was associated with true AF recurrence after RF ablation, it was not a predictor of true AF recurrence after CB ablation.
